# Can Selected Parameters of Brain Injury Reflect Neuronal Damage in Smoldering Multiple Sclerosis?

**DOI:** 10.3390/diagnostics14171993

**Published:** 2024-09-09

**Authors:** Natalia Niedziela, Maria Nowak-Kiczmer, Lina Malciene, Mariusz Stasiołek, Jolanta Zalejska-Fiolka, Zenon P. Czuba, Jacek T. Niedziela, Jarosław Szczygieł, Michał Lubczyński, Monika Adamczyk-Sowa

**Affiliations:** 1 Department of Neurology, Faculty of Medical Sciences in Zabrze, Medical University of Silesia in Katowice, Ul. 3-go Maja 13-15, 41-800 Zabrze, Poland; marianowak185@gmail.com (M.N.-K.); jaroslawszczygiel@op.pl (J.S.); m.adamczyk.sowa@gmail.com (M.A.-S.); 2 Klaipeda University Hospital, Lithuanian University of Health Sciences, 44307 Kaunas, Lithuania; lmalciene@gmail.com; 3 Department of Neurology, Medical University of Lodz, Ul. Kopcińskiego 22, 90-419 Łódź, Poland; mariusz.stasiolek@umed.lodz.pl; 4 Department of Biochemistry, Faculty of Medical Sciences in Zabrze, Medical University of Silesia in Katowice, Ul, Jordana 19, 41-808 Zabrze, Poland; jzalejskafiolka@sum.edu.pl; 5 Department of Microbiology and Immunology, Faculty of Medical Sciences in Zabrze, Medical University of Silesia in Katowice, Ul. Jordana 19, 41-808 Zabrze, Poland; zczuba@sum.edu.pl; 6 3rd Department of Cardiology, Faculty of Medical Sciences in Zabrze, Medical University of Silesia in Katowice, Silesian Centre for Heart Disease, Ul. M.C. Sklodowskiej 9, 41-800 Zabrze, Poland; jniedziela@sum.edu.pl

**Keywords:** NF-H, GFAP, S100B, UCHL1, interleukins, smoldering multiple sclerosis, neurodegeneration, neuroinflammation

## Abstract

Background: Inflammatory demyelination and impaired recovery processes result in permanent neurodegeneration and neurological disability in patients with multiple sclerosis (MS). In terms of smoldering MS, chronic neuroinflammation develops in the early period of the disease and leads to confirmed disability accumulation. There is a great need to identify biomarkers of neurodegeneration and disease progression. Methods: A single-center prospective observational study was performed. The median age of the patients was 40 (31–52) years. Women comprised 64% of the study population. We evaluated the concentrations of the parameters of brain injury (NF-H, GFAP, S100B and UCHL1) in the cerebrospinal fluid (CSF) and the selected interleukins (ILs) in serum of 123 relapsing–remitting MS (RRMS) and 88 progressive MS (PMS) patients. Results: The levels of GFAP, S100B and UCHL were higher in the PMS group than the RRMS group, in contrast to the levels of NF-H. We observed a positive correlation between the selected pro-inflammatory cytokines and the parameters of brain injury. The Expanded Disability Status Scale (EDSS) score increased with GFAP and NF-H levels and was correlated with the selected ILs. The concentrations of S100B, UCHL1 and NF-H reflected the duration of MS symptoms. Conclusions: The levels of brain injury parameters in the CSF and the selected serum ILs in MS patients seem to be promising biomarkers to determine neurodegeneration and neuroinflammation in smoldering MS. Further studies are warranted in this respect.

## 1. Introduction

Multiple sclerosis (MS) is a chronic immune-mediated disease of the central nervous system (CNS) characterized by inflammation, demyelination, axonal damage and neurodegeneration [1,2]. In young adults, MS is the leading cause of disability, affecting 2.8 million people worldwide [3]. This heterogeneous disorder varies in presentation, clinical course and prognosis.

The pathological process in MS is triggered by the infiltration of autoreactive B and T lymphocytes that represent identifiable factors responsible for acute peripheral inflammation. It manifests as focal inflammatory lesions in the CNS and relapses [4]. When peripheral immune cells infiltrate the CNS, the formation of perivascular demyelination and neuroaxonal degeneration is reported [5]. On the other hand, pro-inflammatory microglia, resident B cells and macrophages are responsible for chronic CNS neuroinflammation, which develops in the early period of the disease and leads to confirmed disability accumulation [6]. Based on the most recent concept of smoldering MS, the pathological process of MS is the result of acute peripheral neuroinflammation and chronic neuroinflammation, but focal inflammatory lesions in the CNS are secondary to the loss of axons and neurons from which myelin antigens are released [7]. Demyelination activates astrocytes and the formation of gliotic scars. At the same time, partial remyelination is observed by the activation of oligodendrocyte progenitor cells [8]. The presence of inflammatory demyelination and impaired recovery processes result in permanent neurodegeneration and neurological disability [9]. 

Clinically isolated syndrome (CIS) refers to the first clinical CNS demyelinating event lasting at least 24 h, possibly preceding a diagnosis of MS. Most MS patients are affected by relapsing–remitting MS (RRMS), with transition to secondary progression manifested as increasing disability (SPMS) [10]. In about 20% of MS patients, the progressive stage is observed from the first symptoms and is defined as primary progressive MS (PPMS) [11]. Together, SPMS and PPMS are referred to as progressive MS (PMS). On the other hand, disability progression independent of relapse activity typical of PMS can also occur in RRMS [12]. Axonal and neuronal loss results in progressive and irreversible disability in the early stages of the disease [13,14]. In the context of smoldering MS, clinical MS phenotypes slowly transit from one phase to another, and there is a continuum of relapsing and progressive phenotypes of MS. Patients experience neuroinflammation and neurodegeneration throughout the whole disease course. Therefore, the accurate identification of specific biomarkers of MS diagnosis and phenotyping is challenging. 

Chronic inflammation results in an imbalance between damage and the functional reserve of the brain [15]. Increased concentrations of the parameters of brain injury reflect neuronal damage. Many molecules, including neurofilaments (NFs), glial fibrillary acidic protein (GFAP), calcium-binding protein (S100B) and ubiquitin C-terminal hydrolase (UCHL1), have been investigated as potential biomarkers of neurodegeneration in MS. GFAP is the major intermediate cytoskeletal protein expressed primarily in the cytoplasm of astrocytes. It is released from astrocytes. GFAP reflects astrogliosis and astroglial damage in response to CNS injury [16]. NFs are neuronal cytoskeletal proteins abundant in axons and consist of four subunits, i.e., light-chain neurofilament (NF-L), medium neurofilament (NF-M), heavy neurofilament (NF-H) and α-internexin. These molecules occur subsequently in the CSF during neuroaxonal injury [17]. Prognostic values of GFAP and NFs as biomarkers in MS have been widely investigated. S100B is an inflammatory molecule and a marker of neuronal damage released mainly from astrocytes. It is also related to impairment of axonal conduction and neuroinflammation [18]. UCHL1 is a neuron-specific deubiquitinating enzyme involved in repairing injured axons and neurons, which also occurs in immune reactions [19]. 

As previously mentioned, immune cell infiltration of the CNS is connected to recurring inflammatory events, while neurodegeneration is associated with the activation of microglia by pro-inflammatory cytokines [20]. Interleukins (ILs) are a group of cytokines that have been suggested to be disease activity biomarkers [21]. All MS phenotypes are characterized by neuroinflammation, and pro-inflammatory ILs mediate inflammatory and immunological processes [22].

Brain and spinal cord magnetic resonance imaging (MRI) and cerebrospinal fluid (CSF) restricted oligoclonal bands (OCBs) play an essential role in the differential diagnosis of MS. However, they do not provide an understanding of the histopathological changes and adequate associations with symptoms. Different studies have been conducted to elucidate markers of neurodegeneration, remyelination, MS prognosis and response to disease immunomodulatory treatment (DMT) and to investigate the mechanisms of progressive decline in MS. 

The aim of this study was to compare the concentrations of the parameters of brain injury between RRMS and PMS patients. Additionally, we assessed correlations between GFAP, NF-H, S100B, UCHL1 and some pro- and anti-inflammatory cytokines. This study was designed to verify the usefulness of non-standard CSF and serum biomarkers in the assessment of brain injury and disease progression in terms of smoldering MS. 

## 2. Materials and Methods 

All consecutive patients with MS were prospectively recruited from the Department of Neurology in Zabrze, Medical University of Silesia, Katowice, Poland. The single-center prospective observational study was performed from October 2023 to March 2024. All patients enrolled in the study were divided into RRMS and PMS (PPMS/SPMS) groups, depending on the clinical type of MS. 

The inclusion criteria were as follows: age ≥18 years; RRMS or PPMS diagnosed according to the McDonald criteria (2017); in the case of SPMS, evidence of progression over ≥3 months; disability progression by 1 step on the EDSS in patients with EDSS ≤ 5.5 or 0.5 EDSS steps in patients with EDSS ≥ 6.0; patients treated with a disease-modifying therapy (DMT) or patients without immunomodulatory treatment before the study; EDSS ≤ 5.0 (RRMS and PPMS); EDSS ≥ 4.0 and pyramidal functional system (FS) 2.0 (SPMS) [23]; Caucasian race; and written informed consent for participation in the study. The exclusion criteria were as follows: contraindications for MRI assessment and lumbar puncture, relapse and steroid therapy during the last six months, neurodegenerative diseases other than MS or other neurological or serious diseases that could affect neurological examination, a history of head injury and stroke during one year before the study, other serious autoimmune disorders, pregnancy and breastfeeding.

All diagnostic procedures were conducted during morning medical visits and included medical history, physical examination, lumbar puncture and a panel of biochemistry blood tests. The clinical stage of MS was determined by an experienced rater using the EDSS. The course of MS, treatment and comorbidities were analyzed based on medical records. The survey questionnaire was prepared to provide additional data on the underlying disease, basic personal data, relapses, the use of DMT and steroid therapy and lifestyle. All patients underwent MRI examination of the brain and cervical and thoracic spine. The MRI images were evaluated by a single radiologist specializing in evaluating examinations of MS patients. The presence of Gd+ lesions was assessed. A lumbar puncture (L3/L4 or L4/L5) was performed to collect cerebrospinal fluid (CSF) for further analysis. 

The concentrations of brain injury parameters, including NF-H, GFAP, S100B and UCHL1, were determined in the CSF using an Invitrogen Brain Injury 4-plex Human ProcartaPlex™ Panel (Thermo Fisher Scientific Inc., Waltham, MA, USA). ProcartaPlex immunoassays are based on the principles of a sandwich ELISA, using two highly specific antibodies binding to different epitopes of one protein to quantitate all protein targets simultaneously using a Luminex instrument. ProcartaPlex assays utilize a Luminex xMAP detection system (Luminex 200). All stages of the analysis were conducted according to the manufacturer’s instructions. Additionally, the levels of the selected serum anti- and pro-inflammatory cytokines were assessed (IL-8, IL-10, IL-11, IL12p70, IL-19, IL-20, IL-22, IL-26 and IL-27p28). Whole blood samples were left to clot at room temperature for 30 min. The supernatant was centrifuged at 1000× *g* for 15 min. Next, serum samples were stored at −80 C until analysis. Before the assessment, the samples were centrifuged for 5 min. A multiplex assay (Bio-PLEX Pro Human Inflammation Panel 1, 37 Plex#171AL001M) was used to measure cytokine levels. The study was approved by the Bioethics Committee of the Medical University of Silesia in Katowice (consent no. PCN/022/KB1/48/I/20).

### Statistical Analysis

Descriptive statistics parameters for continuous variables are presented as median and interquartile range. Qualitative variables are presented as percentage values. The Mann–Whitney U test was used to compare two subgroups. Group homogeneity with respect to the qualitative variables was analyzed by chi-squared test using Fisher’s exact test when the expected frequency table included values <5. The frequencies between the subgroups were compared using contingency tables and the chi-square test. The linear correlations between the variables were calculated using Pearson’s R test for correlation with 95% confidence interval (grey shadows on the plots). A significance level of *p* < 0.05 was adopted. All statistical analyses were performed using R version 4.2.2 (R Core Team [2022]. R: A language and environment for statistical computing. R Foundation for Statistical Computing, Vienna, Austria) and RStudio (RStudio Team [2020]. RStudio: Integrated Development for R. RStudio, PBC, Boston, MA, USA).

## 3. Results

Out of two hundred fifty-two patients, two hundred and eleven subjects with MS were prospectively enrolled in the study. Forty-one did not meet the inclusion criteria; twenty due to relapse and steroid therapy during the last six months, two women because of the breastfeeding and nineteen due to other diseases that could have affected clinical and laboratory results (concomitant neurodegenerative diseases other than MS and cardiac and pulmonary disorders). The median age of the whole study population was 40 (31–52) years. Women comprised 64% of the study population. The neurological status of the patients assessed by EDSS score was determined to be 3.50 (2.50, 4.50). Patients on DMT at the time of the study constituted 12.0% of the study group. 

In the whole study group, there were 123 patients with RRMS and 88 with PMS (56 with PPMS and 32 with SPMS). The general characteristics of the study groups are given in Table 1. Patients with PMS were older than RRMS subjects. Significant differences were observed in the total scores measured by the EDSS, in MS duration and in the duration of MS symptoms in PMS patients compared to RRMS subjects. There were more females in the RRMS group than in the PMS group. Relapses and Gd+ lesions in brain MRIs were more prevalent in RRMS patients than in the PMS group. No differences were found in OCBs or elevated IgG in the CSF between RRMS and PMS patients.

### 3.1. Assessment of the Selected Parameters of Brain Injury in the CSF and the Selected Serum Interleukins in MS Patients

The concentrations of GFAP, S100B and UCHL1 in the CSF were higher in PMS patients than in the RRMS group, while the level of NF-H did not differ between these groups. Higher levels of IL-11 and IL-20 were also observed in patients with PMS. However, the concentrations of IL-27p28 were decreased in patients with PMS compared to the RRMS group (Table 2). 

#### 3.1.1. Correlations of the Selected Serum Interleukins with the Selected Parameters of Brain Injury in the CSF in MS Patients

A negative correlation was found between the concentrations of IL-27p28 and GFAP (R = −0.29, *p* < 0.001), while IL-10 correlated negatively with NF-H (R = −0.45, *p* < 0.001), S100B (R = −0.38, *p* < 0.001) and UCHL1 (R = −0.28, *p* < 0.001). The levels of IL-8 and IL-11 increased with the concentrations of GFAP (R = 0.15, *p* = 0.028; R = 0.71, *p* < 0.001, respectively), NF-H (R = 0.4, *p* < 0.001; R = 0.23, *p* < 0.001, respectively) and S100B (R = 0.21, *p* = 0.002; R = 0.26, *p* < 0.001, respectively). A positive correlation was also found between IL-20 and GFAP (R = 0.18, *p* = 0.009) and UCHL1 (R = 0.23, *p* < 0.001), as well as between IL-26 and GFAP (R = 0.22, *p* = 0.002). We did not observe any other correlations between serum ILs and the parameters of brain injury in the CSF in the whole MS cohort.

#### 3.1.2. Correlations of the Selected Serum Interleukins with the Selected Parameters of Brain Injury in the CSF in RRMS and PMS Patients 

**Figure 1:**
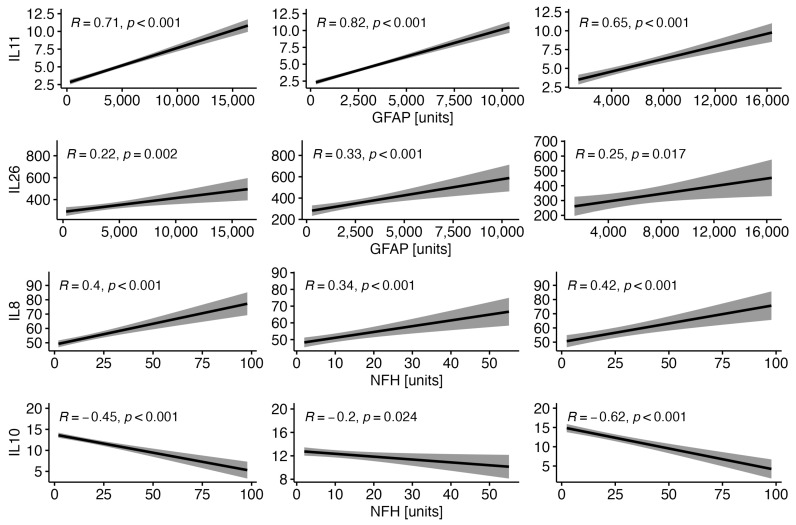
Correlations between the selected serum ILs and the selected parameters of brain injury in the CSF in MS (**left column**), RRMS (**middle column**) and PMS patients (**right column**).

IL-10 negatively correlated with NF-H in RRMS patients and with NF-H, S100B and UCHL1 in PMS subjects. Negative correlations were found between IL27p28 and GFAP and between NF-H in RRMS patients but not in PMS patients. The levels of IL-11 increased with the concentrations of GFAP, NF-H and S100B in RRMS patients, as opposed to PMS patients, in whom the levels of IL-11 increased only with GFAP. Positive correlations were also found between IL-8 and NF-H and S100B in RRMS patients and between IL-8 and NF-H and GFAP in the PMS group. IL-26 was positively correlated with GFAP in RRMS and PMS patients, while IL-20 was positively correlated with GFAP in the RRMS group and with UCHL1 in PMS patients. No other correlations between serum ILs and the parameters of brain injury were found in the CSF in the RRMS and PMS groups. The correlations between the selected serum ILs and the selected parameters of brain injury in the CSF in RRMS and PMS patients are given in Table 3. Some correlations between serum ILs and the parameters of brain injury in the CSF were observed in the whole MS cohort and in patients with RRMS and PMS (Figure 1).

### 3.2. Correlations between EDSS and the Selected Interleukins in MS Patients

EDSS correlated positively with IL-8 and IL-19 in the whole MS cohort (R = 0.18, *p* = 0.008; R = 0.24, *p* < 0.001, respectively) and in RRMS patients (R = 0.21, *p* = 0.019; R = 0.19, *p* = 0.031, respectively), while it correlated positively only with IL-19 in the PMS group (R = 0.7, *p* < 0.001). Negative correlations were noted between EDSS and IL-27p28 in the whole MS cohort (R = −0.31, *p* < 0.001) and in the PMS group (R = −0.38, *p* < 0.001) and between EDSS and IL-10 in the RRMS group (R = −0.41, *p* < 0.001).

### 3.3. Correlations between EDSS with the Selected Parameters of Brain Injury in Patients with MS

**Figure 2:**
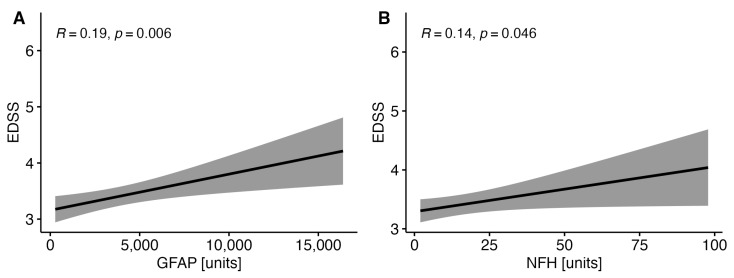
Correlations between EDSS and the concentrations of GFAP (**A**) and NF-H (**B**) in the whole MS cohort.

EDSS correlated positively with the concentrations of GFAP and NF-H in the whole MS cohort (Figure 2). No correlations were found between EDSS and GFAP (R = 0.08, *p* = 0.466) and NFH in the RRMS group (R = 0.08, *p* = 0.366) or in the PMS group (R = 0.36, *p* = 0.34; R = 0.26, *p* = 0.13, respectively). 

### 3.4. Correlations between the Duration of MS Symptoms and the Selected Parameters of Brain Injury in MS Patients

**Figure 3:**
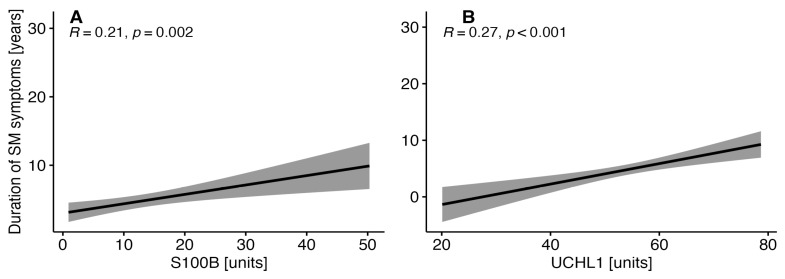
Correlations between the duration of MS symptoms and S100B (**A**) and UCHL1 (**B**) in the whole MS cohort.

The duration of MS symptoms correlated positively with the concentration of S100B in the whole MS cohort (R = 0.21, *p* = 0.002) and in patients with PMS (R = 0.25, *p* = 0.017). The duration of MS symptoms correlated positively with UCHL1 in the whole MS cohort (R = 0.27, *p* < 0.001) and in the RRMS group (R = 0.33, *p* < 0.001). A positive correlation was found between the duration of MS symptoms and NF-H in RRMS patients (R = 0.19, *p* < 0.036). Correlations between the duration of MS symptoms and S100B and UCHL1 in the whole MS cohort are given in Figure 3.

## 4. Discussion

Patients with MS experience neuroinflammation and degeneration during the disease course, which adequately reflects the complexity and heterogeneity of MS pathology. The impairment of the blood–brain barrier, lymphocyte infiltration, activation of microglia, release of pro-inflammatory cytokines with demyelination and chronic neuroinflammation facilitate neuronal loss. Inflammatory lesions are related to secondary neurodegeneration, which is defined as axonal and neuronal loss. Mitochondrial dysfunction, reactive oxygen species, iron accumulation and decreased remyelination capacity also contribute to neurodegeneration and clinical disability in MS patients. Additionally, there is some evidence of the existence of a glymphatic system for the removal of toxic waste products of tissue damage in the CNS. The glymphatic system may be impaired in MS, causing exacerbation of inflammatory and neurodegenerative processes and neurological disability [24]. Considering the above, studies are warranted to identify biomarkers of disease progression. 

In smoldering MS, the pathological process results from the co-existence of acute peripheral inflammation and chronic neuroinflammation in the CNS. The clinical indicator of smoldering MS is progression independent of relapse activity (PIRA), which needs to be distinguished from relapse-associated worsening (RAW) [7]. There is some evidence that PIRA and RAW are common processes leading to disability in RRMS and PMS [12]. Therefore, the determination of markers of acute inflammatory relapse and neurodegeneration in clinical progression is necessary. 

Compared to serums, the CSF is closer to the brain’s extracellular space and contains higher levels of CNS-derived proteins. As a result, it is a potentially better neuronal and glial biomarker reflecting brain injury in MS [25]. 

GFAP is an indicator of hyperplasia of astrocytes following brain injury. Due to its rapid release in axonal degeneration and following astrogliosis, GFAP is considered a CSF biomarker [26]. In our study, higher levels of GFAP in the CSF were observed in PMS patients compared to RRMS. This finding is in line with a meta-analysis conducted by Momtazmanesh et al. that showed higher GFAP levels in the CSF of PPMS patients compared to RRMS subjects [17]. In another meta-analysis of 11 clinical trials comprising 960 MS patients, GFAP levels in the CSF were significantly elevated in MS patients compared to healthy controls. The results indicated no differences in concentrations of GFAP in the CSF between PPMS and SPMS [27]. Therefore, in our study, these groups were combined as PMS. As a result, mean levels of GFAP in the CSF were higher in PMS compared to RRMS patients. In the context of smoldering MS, progressive and irreversible disability caused by axonal and neuronal loss is found in the early stages of the disease, which further suggests a continuum of relapsing and progressive phenotypes of MS (PPMS and SPMS) [13,14]. Kassubek et al. noted elevated levels of GFAP in the CSF at the early stage of RRMS [28]. Furthermore, increased serum levels of GFAP in MS patients [29] and in the CSF of RRMS subjects diagnosed de novo compared to the control group were reported [30]. Högel et al. found elevated serum levels of GFAP in progressive MS and an association with clinical disability [31]. However, earlier studies suggested a mild to moderate relationship between serum GFAP and increased disability [32,33]. The differences in the results of serum GFAP analysis across studies may be related to variations in detection methods. In the past, identification of GFAP in serum was challenging because an ELISA test could not detect such a low concentration. Currently, highly sensitive assays (single-molecule arrays; Simoa) are used to detect GFAP in healthy individuals [25]. 

In our study, correlations were observed between EDSS and GFAP and NF-H in the CSF in the MS cohort, which is in line with an analysis conducted by Ayrignac et al. [34]. Similarly, serum GFAP in PPMS patients did not correlate with the EDSS. However, correlations were reported between GFAP and the Timed 25-Foot Walk test (T25FW) [35]. In smoldering MS, routine neurological evaluation of patients according to the EDSS does not seem to be a sufficiently sensitive method to find neuronal damage resulting in disability progression. Cadavid et al. suggested using the EDSS-Plus, which includes the 9-Hole Peg Test (9HPT) and T25FW, which separate SPMS progressors from non-progressors [36]. In terms of disability progression, further studies related to the correlations between EDSS and GFAP levels are warranted.

GFAP seems to be a useful parameter for auxiliary diagnosis of MS. It is a marker of astrocyte and microglia activation, which are major drivers in PMS. The level of GFAP in the CSF reflects different degrees of damage to astrocytes in different MS phenotypes and allows MS subtypes to be distinguished. In smoldering MS, disability progression begins at diagnosis. Based on our analysis and the findings of other studies, the level of GFAP in the CSF is a promising biomarker for detecting neuronal damage and disability progression in MS patients. 

NF levels in the CSF have been widely investigated in MS. These molecules are intermediate filament neuronal cytoskeletal proteins released due to neuroaxonal injury. NFs are composed of NF-L, NF-H, NF-M and α-internexin or peripherin in the CNS and peripheral nervous system, respectively. In their meta-analysis, Momtazmanesh et al. showed that the level of NF-L in the CSF was significantly higher in MS patients compared to controls. Patients with clinically isolated syndrome (CIS) had also higher NF-L levels in the CSF compared to controls [17]. A useful value of NF-L in the CSF was reported by Bjornevik, who indicated that the levels of NF-L increased six years before the clinical onset of MS [37]. Similarly, the concentration of NF-H in the CSF was increased in RRMS patients diagnosed de novo and in subjects with 5-year RRMS evolution compared to the controls [30,38]. In comparison to NF-L, the role of NF-H in clinical practice is limited [39]. Therefore, we decided to use NF-H in the evaluation of neuronal damage. Compared to subjects with non-inflammatory neuropsychiatric diseases, in patients with CIS, NF-H levels correlated with brain volume and disability during a one-year study [40]. NF-H concentration in the CSF also correlated with EDSS scores, in contrast to NF-L [41]. On the other hand, increased NF-H levels were found to be a predictor of conversion from CIS to clinically definite MS [42]. Increased NF-H concentrations were associated with EDSS score [38] and relapse activity [43,44]. NF-L and NF-H were decreased in the CSF in patients during natalizumab therapy [43]. Studies evaluating the concentrations of NF-L and NF-H in the CSF and serum in MS patients are still lacking. In our study, no differences were found in the level of NF-H in the CSF between RRMS and PMS patients, which is in line with the results of a meta-analysis conducted by Momtazmanesh et al. [17]. Inconsistent findings were noted by Martin et al., who showed higher levels of NF-L in the CSF of RRMS patients compared to PMS [45]. This difference could be explained by confounding factors, including sex, age, DMT, assessment of different subunits or NFs in various body fluids. Some data have indicated that age could be a key determinant of the levels of NF-L and GFAP in the CSF [17,46]. In our study, patients with PMS were older than those with RRMS. Despite this, no differences in the levels of NF-H in the CSF were found between the RRMS and PMS groups. The association between NFs seems to be controversial. Bridel at al. reported a correlation between serum NF-L and age in healthy individuals but not in MS patients [47]. Some studies have also documented a relationship between age and CSF, as well as serum NF-L, in MS [48,49]. Our analysis of the association between the concentration of NF-H in the CSF and age requires further investigations. We found a positive correlation between the duration of MS symptoms and NF-H only in patients with RRMS. In addition, studies on PMS have suggested that DMT can modify the levels of NF-L. Treatment with siponimod in the ASCEND clinical trial and natalizumab in the EXPAND trial reduced serum NF-L levels [35]. 

As previously mentioned, our study found correlations between EDSS and GFAP and NF-H in the CSF in the whole MS cohort but not in patients with RRMS or PMS. Benkert et al. reported that NFs reflected disease activity and were associated with disease progression [50]. However, their potentials were found mainly in RRMS, while the results were inconclusive in PMS patients [51,52]. NFs seem to be a useful biomarker of MS activity because their levels increase during acute demyelination and neuro-axonal injury, reflecting acute relapses. In their meta-analysis, Momtazmanesh et al. noted higher levels of NF-L in the CSF during relapse compared to remission [17]. However, patients in our study experienced no relapses during the last six months before enrollment, which may explain the lack of differences between the RRMS and PMS groups.

NFs are a promising predictor of the conversion of CIS to MS, disease activity and differentiation of MS patients at the early stage of the disease. Additionally, they could be a potential prognostic marker of DMT efficacy. Based on our analysis and the findings of other studies, the application of NFs to determine PIRA in MS patients requires further studies.

S100B is a Ca^2+^ binding protein that is highly expressed in the CNS after injury and plays intracellular and extracellular roles. At physiological levels, S100B promotes cell proliferation and migration and modulates synaptogenesis and neurite outgrowth. Elevated S100B concentrations result in glial activation, exacerbate the inflammatory response and release pro-inflammatory cytokines and stress-related enzymes [53,54]. Higher S100B concentrations trigger the activation of microglia and astrocytes, which results in a release of free-radical NO [55]. Elevated S100B levels were found in the CSF of acute-phase MS patients [56]. In their meta-analysis, Momtazmanesh et al. found that the concentration of S100B was significantly higher in the CSF of MS patients compared to controls [17], which is not in line with the lack of differences observed in the concentrations of S100B in the CSF of patients diagnosed de novo with RRMS compared to the controls in [30]. In our study, the concentration of S100B in the CSF was higher in patients with PMS compared to RRMS. These findings are not in line with the results of another study that found no differences in CSF levels of S100B between RRMS and SPMS patients [57,58]. Additionally, in our study, the duration of MS symptoms correlated positively with the concentration of S100B in the whole MS cohort and PMS patients. Several studies have investigated the levels of S100B in the CSF of RRMS and PMS patients. However, the results were not suitable for statistical analysis. Barateiro et al. showed increased S100B concentrations upon diagnosis with MS. However, different MS phenotypes with various stages of demyelination were considered [55].

Based on our findings and those of other studies, S100B remains a useful biomarker of neuronal damage. Histopathologically, smoldering MS is characterized by chronic active lesions, with elevated S100B levels reported in demyelinated areas and lower expression of its receptor on the rim [18]. We observed differences in the levels of S100B in the CSF between RRMS and PMS patients. Slowly expanding lesions (SELs) reflect chronic active lesions in MRI [59]. Considering the above, S100B may be a promising biomarker of neurological damage, disease-related disability and smoldering MS. 

UCHL1 is a small molecule predominantly expressed in neuroendocrine cells and neurons of the brain and spinal cord. It plays a putative role in the degradation and removal of the selected proteins and in the redox state. UCHL1 is an enzyme involved in repairing injured axons and immune reactions [60]. However, reactive oxygen species, reactive lipids and NO may impair its activity. Loss of UCHL1 function is related to the pathogenesis of brain injury and neurodegeneration [61]. The level of UCHL1 was reported to be increased in serum and the CSF after traumatic brain injury and to be associated with the severity of injury and long-term outcome [19,62]. In our study, differences in the levels of UCHL1 in the CSF were found between RRMS and PMS patients. To date, the role of UCHL1 in MS has hardly been investigated. Górska et al. reported higher plasma UCHL1 concentrations in RRMS patients compared to healthy controls. However, plasma UCHL1 concentrations did not correlate with the age of the patients, the number of relapses within 24 months, EDSS, the number of years from the first MS symptoms and diagnosis [19]. Similarly, our findings indicate no correlation between the EDSS and the level of UCHL1 in the CSF of MS patients or in patients with RRMS or PMS. On the other hand, only the levels of UCHL1 and S100B in the CSF were positively correlated with the duration of MS symptoms in the MS cohort. Our findings are probably related to the neurodegeneration process, which increases with the duration of symptoms. Further studies on the distribution of UCHL1 in the CNS and plasma are warranted to explain the discrepancies.

UCHL1 was recognized as a pro-inflammatory cytokine modifying the immune system in MS [19]. We reported a positive correlation between the level of UCHL1 in the CSF of MS patients and pro-inflammatory IL-20 and a negative correlation between the concentration of UCHL1 and anti-inflammatory IL-10 in the whole MS cohort and in patients with PMS but not with RRMS.

UCHL1 is a potential marker to distinguish MS patients from healthy individuals, allowing for a diagnosis of MS and for PMS patients to be distinguished from RRMS patients. Our study also indicates its role as a marker of disease duration. Studies on the impact of UCHL1 on the course of MS are very limited, and the results obtained to date should be validated with a larger group of patients.

Interleukins (ILs) are a group of cytokines with immunomodulatory properties involved in cell proliferation, migration, adhesion, immune cell differentiation and activation. The human genome encodes over 50 various ILs. Some ILs can have pro- and anti-inflammatory characteristics due to their effects on specific cells [63]. As the molecules of the immune response, they may influence the disease course. In MS pathology, dysregulation between pro- and anti-inflammatory cytokines is observed [64], which results in the increased permeability of the blood–brain barrier (BBB), contributing to the development of neurodegeneration and demyelination in the CNS [65]. 

In our study, we observed negative correlations between the concentrations of NF-H, S100B and UCHL1 in the CSF and serum IL-10 in the whole MS cohort. IL-10 was also negatively correlated with S100B and UCHL1 in the CSF of patients with PMS. Negative correlations were found between IL-10 and NF-H in the whole MS cohort and in patients with RRMS and PMS. This molecule is one of the most significant anti-inflammatory cytokines secreted by Th-2 and inhibits Th-1 in MS patients [66]. Studies have reported that S100B released by activated astrocytes could induce damage by activating NF-kB in the inflammatory response and overexpression of TNF-α and IL-1β. Studies have also demonstrated that S100B could induce microglial pro-inflammatory polarization and inhibit anti-inflammatory polarization. Therefore, we noted negative correlations between anti-inflammatory cytokine IL-10 and the selected parameters of brain injury. Positive correlations were found between S100B and TNF-α, S100B and IL-1β [58], and NF-H and soluble TNF receptor 1 in subacute sclerosing panencephalitis [67]. 

IL-27p28 is another anti-inflammatory cytokine. Different analyses have shown the opposing roles of IL-27 in promoting or inhibiting Th-cell differentiation. As a result, IL-27 has a beneficial role in inhibiting CNS inflammation [68]. In our study, we observed negative correlations of the concentrations of serum IL-27 with the levels of GFAP in the CSF in the whole MS cohort and in patients with RRMS and with NF-H in patients with RRMS. We also found negative correlations between the EDSS and the serum concentration of IL-27 in the whole MS cohort and in patients with PMS.

Our study found positive correlations between the levels of serum IL-8 and GFAP, NF-H and S100B in the CSF in the whole MS cohort. The concentration of IL-8 was also positively correlated with NF-H in the CSF of patients with RRMS and PMS, as well as with the level of S100B in RRMS subjects. This cytokine is known as a chemotactic cytokine involved in inflammation, proangiogenesis and tumor invasion [69]. Data on the association between NF-H and ILs in MS are very limited. Daoud et al. reported on the relationship between elevated NF-H levels and IL-8, with unfavorable outcomes in pediatric patients after severe brain injury [70]. 

IL-11 is associated with the stimulation of differentiation of Th17 lymphocytes and promotes the migration of monocytes, neutrophils and CD4+ lymphocytes to the CNS. Studies reported that IL-11 was the most increased cytokine in serum and the CFS in patients with CIS compared to controls. Furthermore, it was significantly higher during relapse, which suggests its pro-inflammatory properties [71]. In our study, the levels of serum were positively correlated with the concentrations of GFAP, NF-H and S100B in the whole MS cohort and in patients with RRMS, which confirmed the coexistence of neurodegeneration and inflammation in smoldering MS. This cytokine was positively correlated with GFAP in the whole MS cohort and in patients with RRMS and PMS. Moreover, the EDSS was positively correlated with pro-inflammatory serum levels of IL-19 in the whole MS cohort and in patients with RRMS and PMS. Together with IL-20 and IL-22, this pro-inflammatory molecule initiates, sustains and drives the progression of vascular disease [72]. At the same time, we found negative correlations between the selected ILs and the EDSS. 

As a product of Th17 lymphocytes, IL-26 not only helps Th17 cells mitigate infections due to direct antimicrobial activity, but it can also be a driver and effector of inflammation in autoimmune diseases [73]. Therefore, positive correlations were found between serum IL-26 and GFAP in the whole MS cohort and in patients with RRMS and PMS. We also investigated other pro-inflammatory cytokines (IL-12p40, IL-12p70 and IL-22). We did not find other correlations between other cytokines and the selected parameters of brain injury. 

ILs may be potential biomarkers of MS progression and activity and can provide information about the pathogenesis of the disease due to their inflammatory and anti-inflammatory properties. However, it is difficult to draw conclusions regarding ILs and MS because the exact function of each cytokine is complicated by the influence of the producing cell type, the phase of the immune response and the responding cell type. ILs can also have pro- and anti-inflammatory properties, which further complicates their characterization. The explanation as to why only the selected pro-and anti-inflammatory ILs may potentially reflect neuropathology in MS patients in our study remains unclear. 

### 4.1. Limitations

This study has some limitations. First, the group of patients with SPSM was relatively small. Therefore, a comparison between patients with SPMS and PPMS was not performed, and we analyzed the combined group (PMS). However, some differences in the levels of brain injury parameters were found between these subtypes and patients with RRMS. Secondly, some patients were on DMT. To the best of our knowledge, some DMT may potentially be related to decreases in the concentrations of brain injury parameters. Our study is also limited by the lack of healthy controls. However, we did not expect an increase in parameters of brain injury in these groups. As a negative control group, we also did not include patients who suffered from neurological diseases other than MS (patients were prospectively recruited from the Department of Neurology) because it was one of the exclusion criteria to the study. Although GFAP is mainly found in astrocytes of the CNS, there is some evidence of other sites of GFAP expression, including the neurogenic stem cells of the subgranular zone in the hippocampus, enteric glia, nonmyelinating Schwann cells or Muller cells. In addition to this, many nonastrocytic cells outside of the CNS express detectable levels of GFAP, such as stellate cells of the liver, pancreas and vocal fold; lymphocytes; vertebral and tracheal cartilage; and Leydig and Sertoli cells. GFAP can also be detected in tumors (e.g., myoepithelial tumors of soft tissue, salivary gland tumors and astrocytomas) [74]. Additionally, the use of various measurement techniques for the assessment of brain injury parameters and comparison of ILs in the CSF and serum may provide inconclusive results. 

### 4.2. Future Directions

Future studies are warranted to further investigate the applications of glial and neuronal biomarkers and ILs in the clinical treatment of MS patients. Many studies have indicated the role of brain injury parameters in distinguishing MS patients from controls. Determining the value of different biomarkers in the diagnosis of MS, distinguishing various subtypes and assessing neuronal damage and disease progression in terms of smoldering MS seem to be most desirable. Additionally, more studies with age- and sex-matched participants are required to confirm the potential clinical utility of brain injury parameters. Patients of non-European ethnicity could also be included in such analyses. The results of this study should be validated in larger cohorts using standardized measurement techniques for brain injury parameters and considering the CSF, in addition to analysis and neuroimaging.

## 5. Conclusions

In summary, the results of our study of a prospectively recruited group of MS patients suggest that GFAP, NF-H, S100B, UCHL1 and the selected ILs are promising biomarkers of neurodegeneration and neuroinflammation in MS. 

The concentrations of GFAP, S100B and UCHL1 in the CSF were higher in PMS patients compared to RRMS subjects. No differences were found in NF-H levels between PMS and RRMS patients. With the exceptions of IL-12 and IL-22, all investigated pro-inflammatory ILs were positively correlated with all parameters of brain injury in the CSF in the whole MS cohort and in patients with RRMS and PMS. However, various combinations were observed. Similarly, we found negative correlations between serum anti-inflammatory IL-10 and IL-27p28 and the parameters of brain injury in the CSF. Some correlations between serum IL-s and the parameters of brain injury in the CSF were noted simultaneously in the whole MS cohort and in patients with RRMS and PMS. These findings confirmed the coexistence of neurodegeneration and neuroinflammation in smoldering MS.

Furthermore, we found positive correlations between the EDSS and serum pro-inflammatory IL-8 and IL-19 in all subjects. A positive correlation was found between the EDSS and IL-19 in the whole MS cohort and in RRMS and PMS. Additionally, the EDSS score increased with the concentrations of GFAP and NF-H in the whole MS cohort. The levels of S100B, UCHL1 and NF-H reflected the duration of MS symptoms.

In conclusion, based on our findings and previous studies, the levels of GFAP and UCHCL1 in the CSF seem to be useful parameters in the diagnosis of MS. The level of GFAP in the CSF reflects different degrees of damage to astrocytes in various phenotypes of MS. It seems to be the most promising biomarker to detect neuronal damage, changes in the EDSS and disability progression in MS patients and to distinguish subtypes of MS. NFs seem to be a useful biomarker to determine MS activity and a promising predictor for the conversion of CIS to MS and to distinguish MS patients at the early stage of the disease, in addition to being a potential prognostic marker of DMT efficacy. The level of S100B was elevated in demyelinated areas, with lower expression of its receptor on the rim of SELs reflecting chronic active lesions in MRI examination. As a result, S100B may be a promising biomarker of smoldering MS and disease-related disability. However, studies on the impacts of UCHL1 and S100B on the course of MS are very limited, and the findings should be validated with larger cohorts. ILs may be useful biomarkers of inflammation in MS disease progression and activity.

The detection of many correlations between the parameters of brain injury, EDSS and pro-inflammatory ILs in all analyzed groups (the whole MS cohort and RRMS and PMS patients) confirmed the hypothesis that in the context of smoldering MS, clinical MS phenotypes slowly transit from one phase to another and that there is a continuum of relapsing and progressive phenotypes of MS. 

Each of the analyzed parameters has its limitations. However, our study indicates the usefulness of non-standard CSF and serum biomarkers in evaluating brain injury. It suggests that the use of brain injury parameters may be adequate when combined with clinical status, neuroimaging and DMT. Further studies are warranted in this respect. 

## Figures and Tables

**Table 1:** General characteristics of the study groups.

Parameter	RRMS (*N* = 123)	PMS (*N* = 88)	*p* Value
Age (years)	36 (29–41)	52 (47–54)	<0.001
Sex (females)	87/123 (71%)	48/88 (55%)	0.016
MS relapse	30/123 (24%)	0/88 (0%)	<0.001
MS duration (years)	0 (0–1)	1 (0–12)	<0.001
MS symptom duration (years)	1 (0–3)	5 (1–13)	<0.001
EDSS (score)	2.50 (2.00–3.00)	4.50 (4.00–5.00)	<0.001
DMT	18/123 (15%)	8/88 (9.1%)	0.227
Gd+ lesions (brain MRI)	48/123 (39%)	8/88 (9.1%)	<0.001
Gd+ lesions (cervical and thoracic MRI)	12/123 (9.8%)	8/88 (9.1%)	0.871
Elevated IgG in CSF	75/105 (71%)	64/88 (73%)	0.841
OCBs	78/117 (67%)	48/88 (55%)	0.078

EDSS—Expanded Disability Status Scale; RRMS—relapsing–remitting multiple sclerosis; PMS—progressive multiple sclerosis; MS—multiple sclerosis; DMT—disease-modifying therapy; Gd+—gadolinium-enhancing lesions; MRI—magnetic resonance imaging; CSF—cerebrospinal fluid; OCBs—oligoclonal bands.

**Table 2:** Comparison of the selected parameters of brain injury in the CSF and the selected serum interleukins in patients with PMS and RRMS.

Parameter	RRMS (*N* = 123)	PMS (*N* = 88)	*p*-Value
GFAP (pg/mL)	1878 (1260–3055)	3626 (1755–8495)	<0.001
NF-H (pg/mL)	4 (3–4)	4 (3–25)	0.070
S100B (pg/mL)	10 (3–16)	13 (10–17)	0.018
UCHL1 (pg/mL)	54 (48–58)	59 (51–62)	<0.001
IL-8 (pg/mL)	47 (42–59)	49 (40–59)	0.236
IL-10 (pg/mL)	12.9 (9.8–14.7)	12.3 (9.5–16.8)	0.238
IL-11 (pg/mL)	3.51 (2.74–4.77)	4.52 (2.87–7.02)	0.005
IL12p70 (pg/mL)	1.40 (0.80–1.96)	1.40 (0.80–1.40)	0.172
IL-19 (pg/mL)	33 (21–44)	33 (15–44)	0.868
IL-20 (pg/mL)	11.58 (8.46–11.58)	11.58 (10.03–13.13)	<0.001
IL-22 (pg/mL)	27 (22–34)	26 (21–34)	0.440
IL-26 (pg/mL)	348 (150–528)	197 (95–582)	0.225
IL-27p28 (pg/mL)	69 (45–87)	50 (10–64)	<0.001

GFAP—glial fibrillary acidic protein; NF-H—neurofilament heavy chains; S100B—calcium-binding protein B; UCHCL1—ubiquitin C-terminal hydrolase L1; CSF—cerebrospinal fluid; RRMS—relapsing–remitting multiple sclerosis; PMS—progressive multiple sclerosis.

**Table 3:** Correlations of the selected serum interleukins with the selected parameters of brain injury in the CSF in RRMS and PMS patients.

Parameter (pg/mL)	RRMS						PMS					
	IL-10	IL-27p28	IL-8	IL-11	IL-20	IL-26	IL-10	IL-27p28	IL-8	IL-11	IL-20	IL-26
GFAP	-	R = −0.31 *p* < 0.001	-	R = 0.82 *p* < 0.001	R = 0.4 *p* < 0.001	R = 0.33 *p* < 0.001	-	-	R = 0.32 *p* = 0.002	R = 0.65 *p* < 0.001	-	R = 0.25 *P* = 0.0017
NF-H	R = −0.2 *p* = 0.024	R = −0.2 *p* = 0.024	R = 0.34 *p* = 0.001	R = 0.18 *p* = 0.047	-	R = −0.22, *p* = 0.014	R = −0.62 *P* < 0.001	-	R = 0.42 *p* < 0.001	-	-	-
S100B	-	-	R = 0.18 *p* = 0.042	R = 0.34 *p* < 0.001	-	-	R = −0.55 *p* < 0.001	-	-	-	-	-
UCHL1	-	-	-	-	-	-	R = −0.59 *p* < 0.001	-	-	-	R = 0.34 *p* = 0.001	-

GFAP—glial fibrillary acidic protein; NF-H—neurofilament heavy chains; S100B—calcium-binding protein B; UCHCL1—ubiquitin C-terminal hydrolase L1; CSF—cerebrospinal fluid; RRMS—relapsing–remitting multiple sclerosis; PMS—progressive multiple sclerosis; IL—interleukin.

## Data Availability

Due to the Personal Data Protection Act, all data are available only upon request from the corresponding author.
